# Segawa syndrome caused by *TH* gene mutation and its mechanism

**DOI:** 10.3389/fgene.2022.1004307

**Published:** 2022-12-08

**Authors:** Yilin Wang, Chunmei Wang, Meiyan Liu, Wuhen Xu, Simei Wang, Fang Yuan, Xiaona Luo, Quanmei Xu, Rongrong Yin, Anqi Wang, Miao Guo, Longlong Lin, Chao Wang, Hongyi Cheng, Zhiping Liu, Yuanfeng Zhang, Fanyi Zeng, Jingbin Yan, Yucai Chen

**Affiliations:** ^1^ Department of Neurology, Shanghai Children’s Hospital, School of Medicine, Shanghai JiaoTong University, Shanghai, China; ^2^ Shanghai Key Laboratory of Embryo and Reproduction Engineering, Key Laboratory of Embryo Molecular Biology of National Health Commission, Shanghai Institute of Medical Genetics, Shanghai Chlidren’s Hospital, School of Medicine, Shanghai JiaoTong University, Shanghai, China

**Keywords:** TH tyrosine hydroxylase deficiency, dopa-responsive dystonia, tyrosine hydroxylase gene, high-throughput sequencing, guanosine triphosphate cyclic hydrolase

## Abstract

Dopa-responsive dystonia (DRD), also known as Segawa syndrome, is a rare neurotransmitter disease. The decrease in dopamine caused by tyrosine hydroxylase (*TH*) gene mutation may lead to dystonia, tremor and severe encephalopathy in children. Although the disease caused by recessive genetic mutation of the tyrosine hydroxylase (*TH*) gene is rare, we found that the clinical manifestations of seven children with *tyrosine hydroxylase* gene mutations are similar to dopa-responsive dystonia. To explore the clinical manifestations and possible pathogenesis of the disease, we analyzed the clinical data of seven patients. Next-generation sequencing showed that the *TH* gene mutation in three children was a reported homozygous mutation (c.698G>A). At the same time, two new mutations of the *TH* gene were found in other children: c.316_317insCGT, and c.832G>A (p.Ala278Thr). We collected venous blood from four patients with Segawa syndrome and their parents for real-time quantitative polymerase chain reaction analysis of *TH* gene expression. We predicted the structure and function of proteins on the missense mutation iterative thread assembly refinement (I-TASSER) server and studied the conservation of protein mutation sites. Combined with molecular biology experiments and related literature analysis, the qPCR results of two patients showed that the expression of the *TH* gene was lower than that in 10 normal controls, and the expression of the *TH* gene of one mother was lower than the average expression level. We speculated that mutation in the *TH* gene may clinically manifest by affecting the production of dopamine and catecholamine downstream, which enriches the gene pool of Segawa syndrome. At the same time, the application of levodopa is helpful to the study, diagnosis and treatment of Segawa syndrome.

## Introduction

**FIGURE 1:**
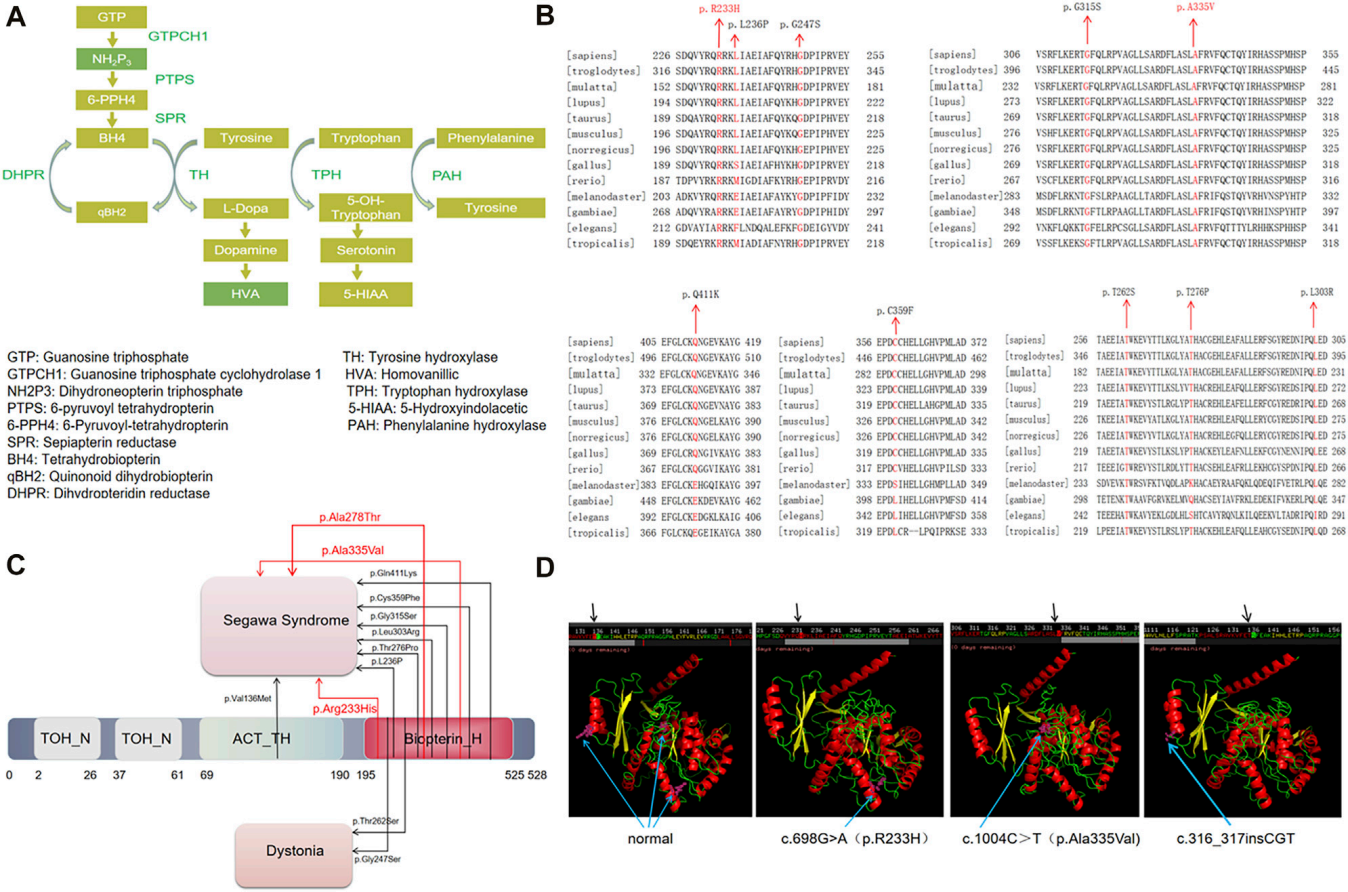
**(A)** The metabolic processes of tyrosine hydroxylase, tryptophan and phenylalanine clarify the pathogenesis of DRD and THD. **(B)** The sequence of proteins corresponding to missense mutation sites in different species. **(C)** TH missense mutation spectrum. Variant listed as red font was discovered in this report. **(D)** The three-dimensional atomic models based on TH protein sequence from wild type and mutation sites generated by iterative threading assembly refifinement server.

Dopa-responsive dystonia (DRD) is a rare neurotransmitter disease with a prevalence of 0.5 per million (Dobricic et al., 2017). It is mainly caused by decreased dopamine synthesis. This condition can manifest itself in several ways, which include, a postural tremor, an abnormal gait, and lower limb dystonia in early childhood or adolescence. Moreover, the condition may also be accompanied by Parkinson’s syndrome (Sun, et al., 2014). In general, the disease mostly afflicts females, and their symptoms have been shown to be more severe than those reported in male patients (Yang, et al., 2018). DRD is thought to be caused by gene mutation in genes that encode enzymes related to dopamine and tetrahydrobiopterin (BH4) biosynthesis. The four genes are: GTP cyclohydrolase 1 (*GCH1*), sepiapertin reductase (*SPR*), 6-pyruvate tetrahydropterin synthase (*PTPS*) and tyrosine hydroxylase (*TH*) (Kuwabara, et al., 2018). Most types of DRD are associated with autosomal dominant mutation in the *GCH1* gene, which result in a GTP cyclohydrolase 1 (GTPCH1) deficiency (Katus and Frucht 2017). Disease caused by a recessive mutation in the tyrosine hydroxylase (*TH*) gene is rare. GCH1 participates in the first and rate-limiting steps of BH4 biosynthesis. BH4 is an important cofactor for tyrosine hydroxylase (TH) to catalyze the conversion of tyrosine to levodopa (Zhang, et al., 2017). Tyrosine hydroxylase is a rate-limiting enzyme for the synthesis of catecholamine, dopamine, epinephrine and norepinephrine (Mak, et al., 2010; Fossbakk, et al., 2014). These compounds are important neurotransmitters involved in the regulation of motor coordination, behavior, learning and memory, sleep-awakening cycle regulation, and endocrine processes and visceral function. If the *TH* gene is mutated, this will impact the levels of catecholamine and its downstream products, such as homovanillic acid (HVA) and 3-methoxy-4-hydroxyphenylethylene glycol (MHPG), which are of great significance for clinical diagnosis (Hou, et al., 2019). The first step in the biosynthesis of catecholamines catalyzed by TH is the conversion of tyrosine to 3,4-dihydroxyphenylalanine (l-DOPA) (van den Heuvel, et al., 1998). The most significant neurodegeneration in patients with dopamine deficiency is in the substantia nigra and striatum dopamine neurons, which are rich in tyrosine hydroxylase (Nagatsu, et al., 2019). Tyrosine hydroxylase deficiency results in a continuous damage to catecholaminergic neurotransmitters, prenatal brain development disorders and postpartum growth disorders (Kuwabara, et al., 2018). Typical clinical manifestations of this deficiency are dystonia, tremors and severe encephalopathy. Based on the genetic mechanisms underlying the occurrence of DRD, levodopa may be an effective drug for its treatment (Figure 1A).

Human *TH* cDNA contains 1921 bp and encodes 528 amino acids. The *TH* gene is located on chromosome 11p15.5 and contains 14 exons (Kobayashi, et al., 1988). Alternative splicing affects the regulation of enzymes through phosphorylation (Grima, et al., 1987). In addition, it contains four domains, namely, the conserved domain Biopterin_H, the ACT-TH domain and two TOH_ N domains at the N-terminus (Hou, et al., 2019). TH is mainly expressed in the brain and adrenal gland (van den Heuvel, et al., 1998). According to the gnomAD database, the frequency of *TH* mutation was 0.84‰ in Chinese individuals. However, in northern China and southern China, the mutation frequency is 1.034‰ and is 0.673‰, respectively, while it is 0 in Japan and Singapore. We reviewed the related literature on the *TH* gene and found that there are 67 significant mutation sites, of which 54 are speculated to be related to DRD, including 17 nonsense mutations and 26 missense mutations. Among them, the c.698G>A (p.Arg233His) mutation may be located in the enzyme active site, as this mutation is associated with only a moderate decrease in solubility and thermal stability, and negatively impacts kinetic activity (Fossbakk, et al., 2014). The c.698G>A (p.Arg233His) mutation site was the first homozygous mutation reported by van den Heuvel LP et al. (van den Heuvel, et al., 1998), and the incidence of this locus is very low, approximately 1/100000000.

## Materials and methods

Contingent upon signing an informed consent form, we collected 2 ml of venous blood from four patients with DRD and their parents. Genomic DNA was extracted from the samples and analyzed following next-generation sequencing (NGS) protocol. The genomic DNA was then treated using ultrasound and hybridized with an array *via* capture by Roche NimbleGen 2.0 probe sequences to obtain an exon DNA library. An Illumina TruSeq DNA sample preparation kit (Illumina, Inc., CA, United States) was used to obtain a precapture library and laser capture microdissection-polymerase chain reaction (PCR) was performed for amplification. The enrichment and size distribution of the library were measured *via* quantitative polymerase chain reaction (qPCR), and its concentration was determined by an Agilent DNA 1000 chip. We sequenced the samples on a HiSeq 2500 (Illumina, Inc., CA, United States) and processed the original image files with BclToFastq (Illumina, Inc., CA, United States). Subsequently, we analyzed the genotypes with a quality score ≥20 (Q20). We used the Burrows-Wheeler Alignment tool (version 0.7.15) to read the sequence data for comparison with the human reference genome 19 (UCSC Genome Browser). Samtools company (http://samtools.sourceforge.net/) and Pindel analysis software (http://gmt.genome.wustl.edu/packages/pindel/) were used to analyze single nucleotide polymorphisms (SNPs) and sequence indices. Finally, we screened the common mutations according to the frequency of exon integration database (ExAC) (minor allele frequency [MAF] < 0.01). The DNA samples obtained from the patients and their parents were confirmed by PCR and Sanger sequencing, and variants identified by whole exome sequencing were determined.

### Protein function prediction of gene mutations

To predict the effects of amino acid changes, we used SIFT (http://provean.jcvi.org/index.php), Polyphen2 (http://genetics.bwh.harvard.edu/pph2/), and Mutation Taster (http://www.mutationtaster.org/). Polyphen2 prediction scores range from 0 to 1, and the prediction results are divided into “probably damaging”, “possibly damaging” and “benign”. If the score is close to 1, it is predicted that the pathogenicity will be greater after amino acid changes. When we use the SIFT/PROVEAN score, if the score is lower than -2.5, then the variation is predicted to be harmful; if the score is greater than -2.5, the variation is neutral. When using Mutation Taster for prediction, the closer the score is to 1, the more likely it is to cause disease.

### Conserved sequence analysis

We analyzed 10 missense mutations using the Human Dec. 2013 (GRCh38/hg38) Assembly UCSC Genome browser.

### Protein structure prediction with the iterative threading assembly refinement (I-TASSER) server for missense mutations

The I-TASSER-Suite pipeline includes four basic steps: thread template identification, iterative structure assembly simulation, model selection and refinement, and structure-based function annotation. The server is located at http://zhanglab.ccmb.med.umich.edu/I-TASSER.

### Real-time qPCR (RT-qPCR) analysis of *TH* gene expression

We used a QIAGEN RNA Preparation Kit (Qiagen Inc., Germany) to extract total RNA from four patients and their parents. cDNA from these samples was subjected to reverse transcription and synthesized using a PrimeScript™ Strand cDNA Synthesis Kit/RT Master Mix (Takara Shuzo Co. Ltd., Japan). Real-time qPCR was conducted using SYBR Premix Ex Taq II (Tli RNase H plus) (Takara Shuzo Co. Ltd., Japan) in LightCycler^®^ 96 Instruments (Roche lnc., AG Schweiz) according to the manufacturer’s protocols. We then used the following primers and double-labeled probes to detect *TH* gene expression: forward primer: GCT​GGA​CAA​GTG​TCA​TCA​CCT​G. reverse primer: CCT​GTA​CTG​GAA​GGC​GAT​CTC​A. After initial denaturation at 95.0°C for 3 min, the reaction was cycled 35 times using at 95.0°C for 30 s and 60.0°C for 45 s. The housekeeping gene *GAPDH* was used as a relative quantity. Each dataset consisting of transcription levels was generated from triplicate studies, and the data are presented as the mean ± SEM. Statistical analyses of qPCR data were performed for comparison of the means of samples using the 2^−ΔΔCT^ method. Asterisks denote statistically significant differences (Student’s t test, **p < 0.05, ***p < 0.01).

## Results

### Clinical data: Clinical manifestations of the patients

We analyzed seven cases with gene damaging variants, and all the children had the clinical manifestations (Table 1). After treatment with Levodopa, some patients’ symptoms were significantly improved, and some patients experienced side effects.

**TABLE 1:** Clinical features of the seven patients.

	Patient1	Patient2	Patient3	Patient4	Patient5	Patient6	Patient7
Sex	Male	Male	Female	Female	Male	Male	Female
Age at examination	6 months	3 years old	2 years old	6 months	4 years old	9 months	8 months
Age at onset	3 months	2 years old	1 years old	2 months	3 months	8 months	5 months
Zygotic state	Compound heterozygosity	homozygote	homozygote	Compound heterozygosity	Compound heterozygosity	homozygote	Compound heterozygosity
Gene mutation	Exon1-13del and c.698G>A (p.Arg233His) in TH	c.698G>A (p.Arg233His) in TH	c.698G>A (p.Arg233His) in TH	c.698G>A (p.Arg233His) and c.1004C>T (p.Ala335Val) in TH	c.738–2A>G and c.316_317insCGT	c.698G>A (p.Arg233His) in TH	c.698G>A (p.Arg233His),c.-19-u25C>T and c.832G>A (p.Ala278Thr) in TH
Family history	No	No	No	No	No	No	The child’s cousin has a history of epilepsy
Dystonia and its nature	Whole body paroxysmal involuntary shaking	No significant changes in muscle tone	Muscular tone was increased with hyperreflexia	Whole body paroxysmal involuntary shaking, knee hyperreflexia, tendon hyperreflexia	Whole body paroxysmal involuntary shaking, knee hyperreflexia	Whole body paroxysmal involuntary shaking	Whole body paroxysmal involuntary shaking, Unstable muscle tone, (high when excited)
Posture or movements	Unsteady head up, won’t take the initiative to grab things	unstable siting alone	Walking on tiptoe, unstable standing, unstable upper limb holder and slight wobble	Unsteady head up, won’t turn over, won’t take the initiative to grab things	Unsteady head up, cannot speak, cannot understand instructions, and cannot sit alone	No abnormalities	Unsteady head up, won’t take the initiative to grab things,won’t turn over, Poor chasing of light and objects, lack of concentration
cMRI	No abnormalities	No abnormalities	High signal intensity in the bilateral radiation corona and paraventricular speckled T2FLAIR	Enlargement of bilateral ventricles and slightly wider extrafrontal space	No abnormalities	No abnormalities	A small amount of bleeding under the dura mater of the left frontal area
Response to levodopa		Good	Good	Moderate	Poor		Good

Case 1 was a six-month-old boy with the *TH* gene compound heterozygous mutation Exon1-13 del (including the upstream INS gene of Exon1) from the mother and c.698G>A (p.Arg233His) from father. This patient was admitted due to paroxysmal involuntary whole-body shaking that had been occurring for 3 months. Despite the tremors occurring on a daily basis, the child never became unconscious. However, child’s psychomotor development lagged behind, manifesting itself in poor sound pursuit and an unstable head. Moreover, the patient showed no initiative to grab things and could not sit upright without support. Video electroencephalogram (VEEG) and a cranial MRI examination showed no abnormalities. The child did not take medication and died of heart disease at the age of 10 months.

Case 2 was a three-year-old boy with homozygous mutation c.698G>A (p.Arg233His). Both of his parents had heterozygous mutation. His parents are not consanguineous to each other, and have no family history. At the age of 10 months, the child showed retrogression in development and could not sit upright without support. The results of video-EEG and cranial MRI examination showed no abnormalities. The child has been treated with Madopar for 10 months, and his symptoms have improved. He now sits steadily, can walk independently, and has no obvious side effects. The development of sports and intelligence needs to be followed up in the future.

Case 3 was a two-year-old girl with the homozygous mutation c.698G>A (p.Arg233His) whose lower limbs were affected. Both of her parents had a heterozygous mutation. This patient was admitted for 10 months because of an unstable gait, accompanied by various related symptoms, such as slow movement of both upper limbs, unstable holding with shaking, unstable sitting, an inability to walk without support, toe walking, drunken movements, and easily falling. A physical examination revealed high muscle tension, muscle strength IV+, bilateral knee reflex and hyperreflexia of the Achilles tendon. The child has a history of growth retardation. Specifically, the child could only raise her head at 6 months old, sit alone at 11 months, and walk without support at 20 months. Cranial MRI showed high signal intensity in the bilateral radiation corona and paraventricular speckled T2FLAIR. At present, the child has been treated with vitamin B1, B6, and B12. Additionally, the child was given oral Madopar without rehabilitative treatment. The child’s symptoms, which included unstable standing, walking on tiptoes, and shaking disappeared. She could walk normally without any obvious side effects.

Case 4 was a six-month-old girl that was admitted to the hospital because of limb trembling. The patient had a compound heterozygous mutation; c.1004C>T (p.Ala335Val) from the mother and c.698G>A (p.Arg233His) from the father. When she was 3 months old, she developed a right-eye squint to the left, limb trembling with head turning to the left, upper limb ankylosis, and lower limb paralysis, lasting for several seconds and occurring more than 10 times a day. The child can chase sound, chase objects, and laugh. However, the child is still unable to turn over, sit upright without support, and she also exhibits lagging growth and development. A physical examination revealed the following abnormalities: a wide eye distance, a narrow eye fissure, and a low ear position. Muscle strength and tension were found to be normal. However, bilateral knee hyperreflexia and Achilles tendon hyperreflexia was observed. Cranial MRI showed an enlargement of bilateral ventricles and a slightly wider extrafrontal space. EEG and other examinations revealed no abnormalities. After oral administration of Madopar (1.5 mg/kg/d), supplementation with vitamin B1, B6, B12, and rehabilitative treatment, the girl’s number of seizures decreased, shifting from being frequent to occasional. The long-term prognosis of this disease requires follow-up.

Case 5, a four-year-old boy, was admitted for more than 3 years due to lagging development and limb trembling during exertion. This patient had a compound heterozygous mutation c.316_317insCGT from the mother and c.738–2A>G from father. The c.316_317insCGT mutation site was not reported in the HGMD Pro database. From an early age, the child was found to have paroxysmal performance, such as sudden upward flipping of the eyes and stiffness of both upper limbs. After these symptoms lasted for approximately half an hour, a fixed gaze, slow response, salivation, and intermittent gnashing of teeth were observed, which lasted for approximately 7-8 h or even 10 h. The child was mentally normal after the attack. The average attack occurs every 3 days. At present, children cannot sit upright without support, swallows poorly, and can only consume a fluid to semifluid diet. The child cannot speak and cannot understand instructions. However, the muscle strength and muscle tension of the child were normal. No abnormality was found in EEG examination and cMRI examination. His mother was given fetal protection treatment due to low progesterone during pregnancy, but the details of this treatment are unknown. The child was delivered by cesarean section at 39 weeks of pregnancy, the amniotic fluid was contaminated at birth, there was no history of rescue, and the birth weight was 3700 g. Other hospitals have given Madopar (2 mg/kg/d) and levodopa to the child. The symptoms of the child did not improve, the convulsions intensified, the number of shakes increased (their duration was prolonged), consciousness was unclear during the attack, and the child also exhibited an unnatural facial expression during the attack. The drug dosage was gradually reduced afterwards, and the child is currently not taking Madopar.

Case 6, a nine-month-old boy with homozygous mutation c.698G>A (p.Arg233His), was admitted for more than 1 month due to involuntary shaking. Both of his parents had heterozygous mutation. With no obvious inducement, the child exhibits whole body involuntary tremor, which is obvious when holding an object or experiencing emotional agitation; the tremor can be relieved for a few seconds, and the attack frequency is uncertain. The parents of the patient are not consanguineous to each other, and have no family history. The video-EEG, cMRI examination, muscle enzyme, lactic acid, and blood ammonia results were normal. The child has not yet been treated with any medication.

Case 7, an eight-month-old girl with compound heterozygous mutation c.698G>A (p.Arg233His), c.-19-u25C>T and c.832G>A (p.Ala278Thr). When the inheritance of the mutation sites is concerned, she inherited c.698G>A (p.Arg233His) and c.-19-u25C>T from the mother and c.832G>A (p.Ala278Thr) from the father. Physically, this patient exhibited an unstable head, an inability to turn over, and developmental delays. Moreover, she was not able to actively grasp objects. These clinical manifestations were accompanied by limb tremors, an upward movement of eyes, backward head tilts, and an abnormal muscle tone (which increased during emotional excitement). EEG examination of the child was normal. MRI of the head showed a small amount of bleeding under the dura mater of the left frontal area. The child is currently being treated with Madopar, and her symptoms have improved significantly. Her head is now stable, and she can actively grasp objects.

### Gene sequencing analysis

We identified five *TH* gene mutation sites, c.698G>A (p.Arg233His), c.1004C>T (p.Ala335Val), c.738–2A>G, and c.316_317insCGT and c.832G>A (p.Ala278Thr). Among them, c.316_317ins CGT and c.832G>A (p.A278T) were not reported in the ExAC and 1000G databases (MAF = 0). The variant classification guidelines of the American College of Medical Genetics and Genomics (ACMG) indicated that the mutation site c.698G>A (p.Arg233His) is associated with pathogenicity, while sites c.1004C>T (p.Ala335Val), c.832G>A (p.Ala278Thr) and c.738–2A>G sites are classified as likely pathogenic variations.

The results of Sanger sequencing revealed the possible pathogenic variants (NM_199,292.2) of the TH gene: c.698G>A (p.Arg233His), c.1004C>T (p.Ala335Val), c.738–2A>G and c.316_317insCGT. We speculate that these variants may affect the normal function of the *TH* gene.

### Protein function prediction of the gene mutations

**FIGURE 2:**
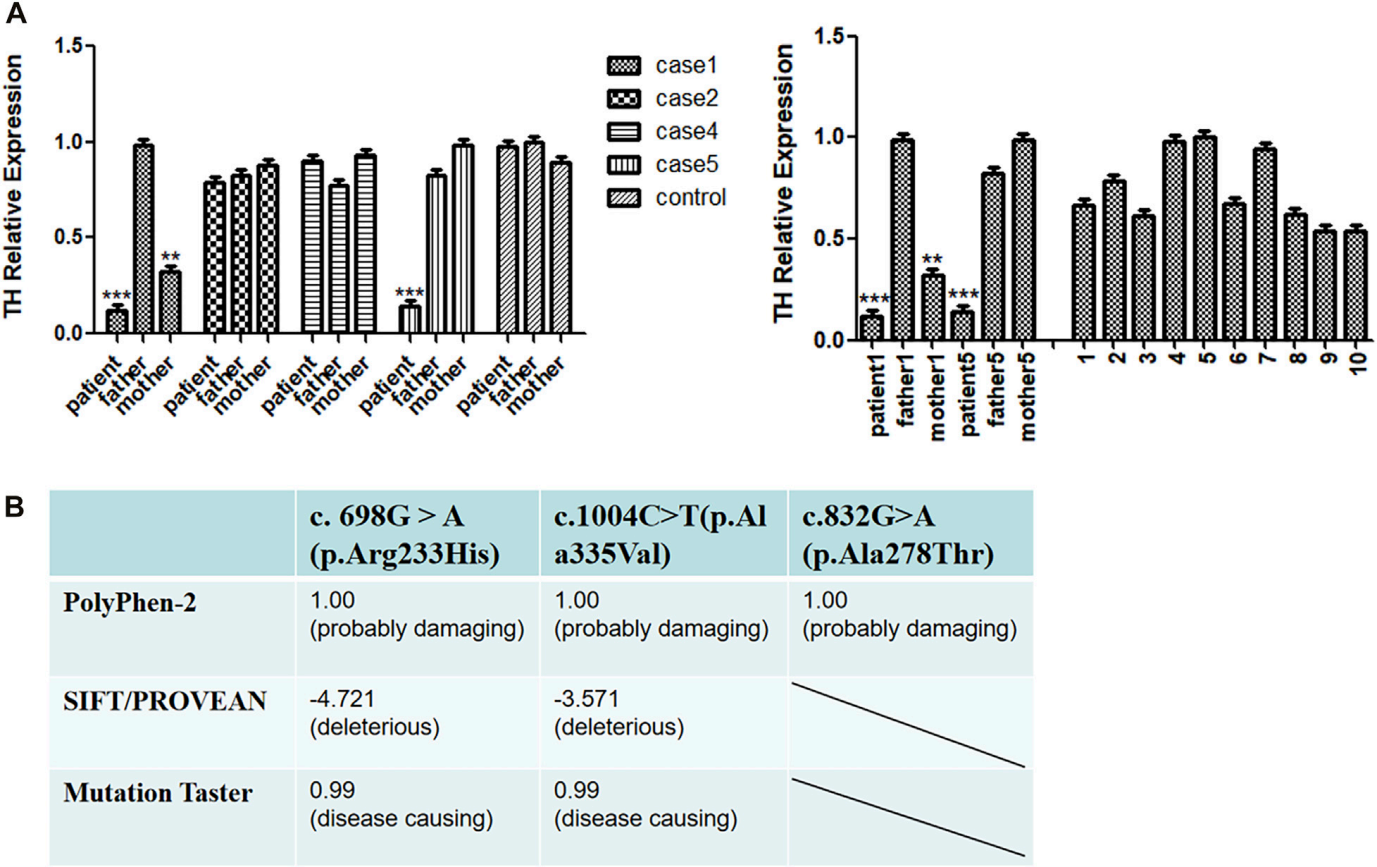
**(A)** Real-time qPCR (RT-qPCR) analysis of TH gene expression. **(B)** Pathogenic scores or protein function prediction results of mutation sites.

The missense mutations c.1004C>T (p.Ala335Val), c.832G>A (p.Ala278Thr) and the reported c. 698G > A (p.Arg233His) mutation of the *TH* gene were predicted by SIFT/PROVEAN, Polyphen-2 and Mutation Taster network software. The results all indicated pathogenic variations (Figure 2B). The mutation site c.316_317insCGT may result in the addition of an amino acid to the amino acid sequence, causing a change in the three-dimensional structure of the protein, and thus a change in protein function.

### Conservation of *TH* gene missense mutations and TH domains

Using the Human Dec. 2013 (GRCh38/hg38) Assembly UCSC Genome browser, we analyzed 10 missense mutations associated with Segawa syndrome which have been reported to date (we focused on the TH protein). We found that all the mutations were in conserved sequences (Figure 1B): c.698G>A (p.R233H), c.710T>C (p.L236P), c.742G>A (p.G247S), c.943G>A (p.G315S), c.1004C>T (p.A335V), c.1234C>A (p.Q411K), c.1079G>T (p.C359F), c.785C>G (p.T262S), c.826A>C (p.T276P), and c.908T>G (p.L303R). These mutation sites are located in the Biopterin_ H domain of the conserved region of the TH protein (Figure 1C). This result shows that the Biopterin_ H domain plays an important role in the structure and function of mature enzyme proteins, and these mutations seem to disrupt the functioning of TH proteins.

### Protein structure prediction on the iterative threading assembly refinement (I-TASSER) server for missense mutation

Iterative thread assembly optimization (I-TASSER) server generated a three-dimensional (3D) atomic model based on missense mutated protein sequences. The results showed that c.698G>A (p.R233H) and c.1004C>T (p.Ala335Val) were not significantly different from the normal three-dimensional model, but the p. Ala335Val mutation site may be located near the tyrosine hydroxylase active site (Figure 1D).

### Real-time qPCR (RT-qPCR) analysis of *TH* gene expression

Based on the above predictions, we speculate that missense mutations in patients with pathogenic variants may reduce the expression of the *TH* gene and affect the function of tyrosine hydroxylase. To explore this possibility, we used RT-qPCR to compare the transcriptional levels of the *TH* gene in the peripheral blood of four patients and their parents. Because the parents of some patients refused to provide a blood sample, we were unfortunately unable to conduct the RT-qPCR tests on them. We found that the expression of cases 1 and 5 was lower than that of 10 normal controls, and that the expression of the case 1 mother was lower than that of the control group, while no positive RT-qPCR result was obtained for cases 2 and 4 (Figure 2A).

## Discussion

Tyrosine hydroxylase deficiency is an autosomal recessive DRD that was first described by Clayton et al. It is characterized by growth retardation, infant Parkinson’s disease, ophthalmic nerve crisis and autonomic nerve dysfunction. According to the symptoms and age of onset, there are three clinical manifestations of the disease: 1) typical DRD, 2) dopa-responsive infantile Parkinson’s syndrome with motor retardation, and 3) progressive infantile encephalopathy (Brautigam, et al., 1999; Clot, et al., 2009; Willemsen, et al., 2010).

This paper reports the mutation of tyrosine hydroxylase in seven patients. Our results expands the known mutation spectrum of tyrosine hydroxylase. These mutations change amino acids and may have important effects on the solubility, activity and stability of the enzyme, thus affecting its function.

Patient one had paroxysmal involuntary whole body shaking, backward psychomotor development, unstable head, poor sound pursuit and did not take the initiative to grab things. This patient did not take medication. *TH* gene compound heterozygous mutation Exon1-13 del (including upstream INS gene of Exon1) and c.698G>A (p.Arg233His) were identified. We used IDTxGenExome Capture Reagent (IDT, United States) for whole-exome capture and sequencing on the HiseqX10 (Illumia, United States) platform. We analyzed the conservation of c.698G>A (p.Arg233His) and found that it was located in the conserved sequence and was predicted as a pathogenic variation. In addition, we found three patients harboring c. 698G > A (p.Arg233His) homozygous mutation, and all of them showed staggering, lameness and tiptoe walking. This mutation causes the amino acid at position 233 to change from arginine to histidine. We analyzed the data of 13 patients harboring missense mutations in the *TH* gene and found that 83% of the missense mutations were concentrated in the region encoding the Biopterin_H domain, and were conserved during evolution, indicating that the Biopterin_ H domain plays an important role in protein function.

We performed RT-qPCR to compare the transcriptional levels of the *TH* gene in the peripheral blood of case 1 and his parents. We found that the expression of patient one was significantly lower than that of normal controls. The expression level of the case 1 mother was also lower than that of the normal controls; this pattern could be attributed to the heterozygous mutation Exon1-13 del (including the upstream INS gene of Exon1) carried by the mother. We speculate that the decrease in *TH* gene expression in case 1 was caused by the deletion of exons 1–13. A patient with early-onset PD was reported to have complete deletion of the *TH* allele, while the other allele had no mutation. The patient did not show symptoms of the catecholaminergic system before the diagnosis of Parkinson’s disease at the age of 54, indicating that neurological symptoms of THD may require more than 50% loss of TH activity (Bademci, et al., 2010). Fossbakk et al. performed functional analysis of the c. 698G>A (p.Arg233His) mutation site and reported suppressed enzyme activity, decreased solubility and thermal stability, and inhibition of residual enzyme activity to 14%; notably, the mutation closer to the C-terminus had a serious impact on activity, which may affect the normal functioning of the TH protein. The site was predicted to be destructive by electronic analysis (Fossbakk, et al., 2014). The active site model of TH binding to L-Tyr and BH4 showed that mutation p. Arg233His was located at the active site (Andersen, et al., 2002; Fossbakk, et al., 2014). We speculate that the degree of loss of tyrosine hydroxylase function may also be related to the degree of tyrosine hydroxylase inactivation. Therefore, the mother of case 1 has no related clinical manifestations at present. Based on the above points, we speculate that this site mutation will lead to abnormal tyrosine hydroxylase activity and affect protein function. Because case 1 was untreated and died of heart disease 10 months after birth, we reviewed the relevant literature for a deeper understanding of this outcome. It was reported that mice carrying the *Th* gene mutation (*Th−/−*) died in the uterus due to the failure of cardiovascular system development, but heterozygous *Th*-deficient mice had a normal lifespan. Inactivation of tyrosine hydroxylase bialleles can lead to mid-embryonic lethality in mice: approximately 90% of mutant embryos die of cardiovascular failure between 11.5 and 15.5 days of the embryo. However, when l-DOPA (dihydroxyphenylalanine), the product of the tyrosine hydroxylase reaction, was applied to pregnant female mice, the mutant mice could be completely saved in the uterus. Without further treatment, though, they died before weaning. It has been suggested that catecholamines are essential for fetal development and postnatal survival in mice (Zhou, et al., 1995). We speculate that case 1 died at 10 months old because of the mutation, especially the dopamine deficiency caused by the deletion of exons 1–13. Specifically, this dopamine deficiency affects myocardial contractility and cardiac output, causing circulatory failure. Unfortunately, because of the parents of the patient refused to provide blood sample, we were unable to carry out the RT-qPCR test. The fourth patient showed unstable head raising, no turning over, no active grasping, lagging growth and development, bilateral knee reflex and hyperreflexia of the Achilles tendon. We conducted RT-qPCR experiments and found that the expression level of the patient was only slightly lower than those of their parents and the control group. The c.1004C>T (p.Ala335Val) site is located in the conserved sequence and was predicted by software to be as a pathogenic variation. Variation of this site may lead to changes in protein function, and the c.1004C>T (p.Ala335Val) site may be located near the predicted active site (Sun, et al., 2014). Therefore, we speculate that this site mutation may change the activity of tyrosinase and affect its binding to the substrate, resulting in a decrease in levodopa synthesis. The missense mutations may cause amino acid changes in important functional regions of the protein, thereby affecting the activity of the enzyme. This may be an important cause of the onset of the disease. Thus, *TH* gene mutations in Case 2 and Case 4 are missense mutations, which may not affect gene expression.

The decrease in *TH* gene expression in case 5 may be due to the presumed mRNA decay or mRNA premature stop caused by the intron variant c.738–2A>G and small insertion c.316_317insCGT. It is known that the compound heterozygous mutation may affect the structure and function of the protein through abnormal splicing. This mechanism may affect the production of dopamine downstream and lead to clinical symptoms.

In all cases, the parents of the affected subjects were examined and found to have no neurological symptoms; the absence of such symptoms reflected decreased penetrance of DRD or, in some cases, two allele mutations were required to cause the disease (Pearl, et al., 2007; Camargos, et al., 2008), as confirmed by our RT-qPCR experiment.

In our study, seven patients had varying degrees of psychomotor retardation or tremors. We performed genetic tests to assist in diagnosis, and then all patients were diagnosed with dopamine reactive dystonia. Dopa-responsive dystonia should be distinguished from the following neurological diseases: 1) Cerebral palsy is characterized by abnormal increases in muscle tone and spasm, often accompanied by intellectual disability, convulsion and emotional disorders, no fluctuation of symptoms and no response to dopa preparation. 2) Juvenile Parkinson’s disease rarely occurs in children less than 8 years of age. PET examination shows that the intake of 18F-dopa decreases. Long-term use of dopa preparations requires a gradual increase in dosage, which tends to cause side effects. 3) Hepatolenticular degeneration is often accompanied by liver function damage, intellectual and mental abnormalities, and the Kayser Fleischer ring can be seen in the cornea. 4) The initial symptoms and signs of a few patients with spastic paraplegia are similar to those of DRD. Among our four patients, two patients had increased muscle tone, two patients had tremor, and the motor function of four patients lagged behind that of children of the same age to varying degrees, but there was no liver dysfunction or a corneal Kayser Fleischer ring. Levodopa was effective in treating the three patients. The symptoms of these patients improved to varying degrees, and the effect was significant, without any obvious side effects. One child had a moderate treatment effect of Madopar, and his symptoms improved slightly. However, one child had obvious side effects after treatment, such as increased body tremor, prolonged attack time, confusion, and unnatural facial expressions. This may be related to sudden inactivity and tremors caused by an overdose of Madopar, and the child has now stopped taking medication. We will continue to follow up on the psychomotor development of the children in the future.

## Conclusion

In this study, we reported the clinical and molecular characteristics of a group of unrelated Chinese patients with DRD. Two new *TH* gene mutation sites, c.316_317insCGT and c.832G>A (p.Ala278Thr) were found. The pathogenicity of different gene sites and the possible pathogenesis of the disease were studied through molecular biology experiments, bioinformatics analysis, and a review of relevant literature review. Our study shows that mutation of the *TH* gene may affect the downstream production of dopamine and catecholamine by changing protein structure, function, and enzyme activity; overall, this may lead to dopa-responsive dystonia. DRD is a rare disease, and its rarity has resulted in clinicians having insufficient knowledge of the mechanisms underlying its occurrence. The patients that were the focus of this study illustrate the importance of clinical manifestations and genetic testing. Gene mutation analysis is helpful and necessary for the diagnosis of TH deficiency and has important guiding significance for follow-up treatment. The diagnosis of dopa-responsive dystonia depends on early clinical manifestations, molecular genetic examination, and experimental treatment *via* levodopa. Early treatment can reduce the burden on patients, families, and society. Treatment with levodopa can lead to continuous improvement of motor development. However, long-term neurological treatment requires years of follow-up.

## Data Availability

The original contributions presented in the study are publicly available. This data can be found here: https://www.biosino.org/node/project/detail/OEP003751.
